# Spatial scale changes the relationship between beta diversity, species richness and latitude

**DOI:** 10.1098/rsos.181168

**Published:** 2018-09-19

**Authors:** Rachakonda Sreekar, Masatoshi Katabuchi, Akihiro Nakamura, Richard T. Corlett, J. W. Ferry Slik, Christine Fletcher, Fangliang He, George D. Weiblen, Guochun Shen, Han Xu, I-Fang Sun, Ke Cao, Keping Ma, Li-Wan Chang, Min Cao, Mingxi Jiang, I. A. U. Nimal Gunatilleke, Perry Ong, Sandra Yap, C. V. Savitri Gunatilleke, Vojtech Novotny, Warren Y. Brockelman, Wusheng Xiang, Xiangcheng Mi, Xiankun Li, Xihua Wang, Xiujuan Qiao, Yide Li, Sylvester Tan, Richard Condit, Rhett D. Harrison, Lian Pin Koh

**Affiliations:** 1 School of Biological Sciences, University of Adelaide, Adelaide 5005, South Australia, Australia; 2 Kellogg Biological Station, Michigan State University, Hickory Corners, MI 49060, USA; 3 Key Laboratory of Tropical Forest Ecology, Xishuangbanna Tropical Botanical Garden, Chinese Academy of Sciences, Yunnan 666303, People's Republic of China; 4 Center for Integrative Conservation, Xishuangbanna Tropical Botanical Garden, Menglun, Yunnan 666303, People's Republic of China; 5 Universiti Brunei Darussalam, Jalan Tungku Link, Gadong, Brunei Darussalam; 6 Forestry and Environment Division, Forest Research Institute Malaysia, Kepong, Selangor 52109, Malaysia; 7 Department of Renewable Resources, University of Alberta, Edmonton, Alberta, Canada T6G 2G7; 8 Bell Museum and Department of Plant and Microbial Biology, University of Minnesota, St Paul, MN, USA; 9 School of Ecological and Environmental Sciences, East China Normal University, Shanghai, People's Republic of China; 10 Research Institute of Tropical Forestry, Chinese Academy of Forestry, Tianhe, Guangzhou 510520, People's Republic of China; 11 Department of Natural Resources and Environmental Studies, National Dong Hwa University, Hualien 97401, Taiwan, Republic of China; 12 State Key Laboratory of Vegetation and Environmental Change, Institute of Botany, Chinese Academy of Sciences, Beijing 100093, People's Republic of China; 13 Institute of Ecology and Evolutionary Biology, National Taiwan University, 1 Roosevelt Road, Section 4, Taipei 10617, Taiwan, Republic of China; 14 Key Laboratory of Aquatic Botany and Watershed Ecology, Chinese Academy of Sciences, Wuhan Botanical Garden, Wuhan 430074, People's Republic of China; 15 Faculty of Science, Department of Botany, University of Peradeniya, Peradeniya 20400, Sri Lanka; 16 Institute of Biology, University of the Philippines, Diliman, Philippines; 17 Institute of Arts and Sciences, Far Eastern University, Manila, Philippines; 18 Institute of Entomology, Biology Centre of the Czech Academy of Sciences and Faculty of Science, University of South Bohemia, Branisovska 31, 370 05 Ceske Budejovice, Czech Republic; 19 New Guinea Binatang Research Center, PO Box 604, Madang, Papua New Guinea; 20 BIOTEC, National Science and Technology Development Agency, 113 Science Park, Klongluang, Pathum Thani 12120, Thailand; 21 Guangxi Key Laboratory of Plant Conservation and Restoration Ecology in Karst Terrain, Guangxi Institute of Botany, Chinese Academy of Sciences, Guilin 541006, People's Republic of China; 22 Tiantong National Forest Ecosystem Observation and Research Station, School of Ecological and Environmental Sciences, East China Normal University, Shanghai 200241, People's Republic of China; 23 Center for Tropical Forest Science – Forest Global Earth Observatory, Smithsonian Tropical Research Institute, Washington, DC, USA; 24 Center for Tropical Forest Science – Forest Global Earth Observatory, Smithsonian Tropical Research Institute, Panama, Republic of Panama; 25 Agroforestry Centre, East and Southern Africa Region, 13 Elm Road, Woodlands, Lusaka, Zambia

**Keywords:** β-deviation, ForestGEO, null model, pairwise dissimilarity, tree diversity

## Abstract

The relationship between β-diversity and latitude still remains to be a core question in ecology because of the lack of consensus between studies. One hypothesis for the lack of consensus between studies is that spatial scale changes the relationship between latitude and β-diversity. Here, we test this hypothesis using tree data from 15 large-scale forest plots (greater than or equal to 15 ha, diameter at breast height ≥ 1 cm) across a latitudinal gradient (3–30^o^) in the Asia-Pacific region. We found that the observed β-diversity decreased with increasing latitude when sampling local tree communities at small spatial scale (grain size ≤0.1 ha), but the observed β-diversity did not change with latitude when sampling at large spatial scales (greater than or equal to 0.25 ha). Differences in latitudinal β-diversity gradients across spatial scales were caused by pooled species richness (γ-diversity), which influenced observed β-diversity values at small spatial scales, but not at large spatial scales. Therefore, spatial scale changes the relationship between β-diversity, γ-diversity and latitude, and improving sample representativeness avoids the γ-dependence of β-diversity.

## 1. Introduction

Decreasing species richness from the equator to the poles is one of the best-recognized patterns in ecology [1,2]. This latitudinal pattern in species richness is consistent across different spatial scales, habitats and taxonomic groups [3]. However, latitudinal differences in species co-occurrence still remain a core question in ecology because of the lack of consensus on the patterns of site-to-site variability in species composition (β-diversity) across latitudinal gradients [4–7]. Difficulties in disentangling the variation caused by pooled species richness (γ-diversity) and site-to-site variation in species composition (β-diversity), as well as in the estimation of β-diversity itself, pose challenges to understanding the latitudinal β-diversity patterns.

Null-model approaches have been proposed to account for variation caused by γ-diversity, by calculating the rate of deviation of observed β-diversity from a null-model generated stochastic expectation (hereafter β-deviation), and have been widely used in studies on β-diversity [4,6,8–10]. Although recent studies have criticized the use of null models (see discussion) [7,11,12], they still provide heuristic values that may help understand how non-random (biological) processes structure local communities. A β-deviation of zero indicates that the observed β-diversity is similar to random sampling, while positive β-deviation values reflect species aggregation [6,8]. As the degree of species aggregation is known to increase with grain size [13], we should expect spatial scale effects on β-deviation as well [10,11].

**Figure 1.**
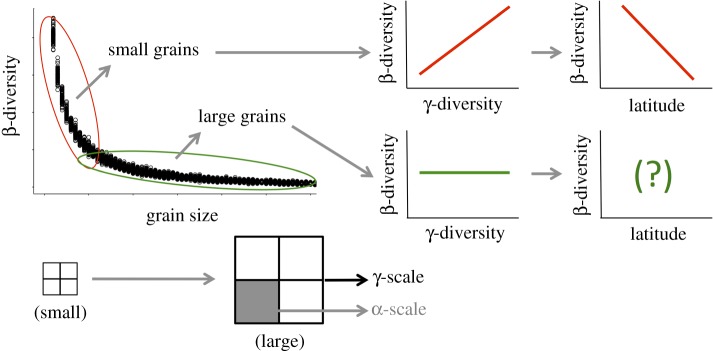
Illustration of the relationship between observed β-diversity and spatial scale (grain size) showing a bi-phasic curve: (1) large variation at small spatial scales, and (2) small variation at large spatial scales. Decreasing γ-diversity with increasing latitude is well known [3], and if β-diversity is correlated with γ-diversity at small spatial scales, we should also expect β-diversity to decline with increasing latitude. However, reliance of β-diversity on γ-diversity is mathematically invalid as long as α-diversity is large and allowed to vary freely with γ-diversity [15]. Therefore, at large spatial scales, we should expect β-diversity not to be reliant on γ-diversity, and the latitudinal β-diversity patterns in such scenarios remain unknown.

The majority of previous studies that examined latitudinal tree β-diversity patterns used small grain sizes to measure α-diversity (less than or equal to 0.1 ha) [5–7,14]. However, studies have demonstrated that β-diversity metrics may risk false conclusions when data are collected using such small grains [15,16], primarily because biodiversity patterns measured at small grains are weaker and more variable [17,18]. Observations show that β-diversity decreases exponentially with increasing spatial scale [19] and can be divided into two segments (figure 1): the first segment where the grain sizes are small and its influence on β-diversity is high, and the second segment where grain sizes are comparatively large and its influence on β-diversity is low (figure 1). Steeper slopes in the first segment can be caused by sampling at small grains that result in artificially lower local (*α*) diversity and higher *γ*:*α* ratios (β-diversity; statistical Type I errors). A lower influence of α-diversity results in the correlation between β- and γ-diversity [20]. This potentially prevents accurate estimation of β-diversity, especially when γ-diversity varies with environmental gradients such as elevation and latitude [6,21]. Previous studies have shown that the influence of γ-diversity on β-diversity decreases with increasing grain size [6,22] and changes β-diversity patterns across broad-scale ecological gradients [22]. The largest grain size in the previous studies was 0.1 ha [22].

In this study, we compare the relationship between β-diversity, γ-diversity and latitude at multiple spatial scales. First, we use tree census data from two 50 ha plots to determine: (i) the sensitivity of β-diversity to grain size and (ii) if the null-model generated β-deviation is also sensitive to grain size. Second, we use tree census data from 15 plots (greater than or equal to 15 ha) along a latitudinal gradient in the Asia-Pacific region to assess: (iii) if the relationship between β-diversity and latitude changes with increasing grain size and (iv) if the relationship between the null model generated β-deviation and latitude remains similar at all grain sizes.

## 2. Methods

### 2.1. Sensitivity of β-diversity

We compared the effects of grain size on classical multiplicative β-diversity and null-model generated β-deviation using woody-plant data from a 52 ha (1040 m × 500 m) forest plot in Lambir Hills National Park, Sarawak, Malaysia (40°186′ N, 114°017′ E; elevation: 104–244 m) and a 50 ha forest plot on Barro Colorado Island (BCI), Panama (9°154′ N, 79°846′ W; elevation: 120–160 m). All stems with a diameter at breast height (DBH) of 1 cm or greater were identified to species and precisely mapped across the entire area. The Lambir and BCI plots contain more than 350 000 and 200 000 mapped trees (greater than or equal to 1 cm DBH) belonging to *ca* 1200 and *ca* 300 species, respectively [23–27]. All stems that are greater than or equal to 1 cm were identified to species and precisely mapped across the entire area. Nothing is omitted and nearly all individuals are assigned to distinct taxa. The Lambir and BCI plots have been censused approximately every five years since 1991 and 1981, respectively. Our analysis of Lambir and BCI plots is based on the 2007–08 census and 2010 census, respectively. The 52 ha (1040 m × 500 m) Lambir plot was trimmed to 50 ha (1000 m × 500 m) to evenly fit multiple non-overlapping grains ranging from 10 m × 10 m to 150 m × 150 m.

A grain is a sample at the local scale (*α*) and an extent (*γ*) is a set of multiple grains. In this study, each extent had a set of nine grains of varying sizes (10 m × 10 m to 150 m × 150 m), all contained within one of the two 50 ha plots (Lambir and BCI). We chose the first sampling grain randomly and the remaining eight were chosen alongside this in a 3 × 3 matrix design. We then repeated the sampling 25 times for each grain size. We measured α-diversity as the mean species richness of each grain and γ-diversity as the species richness of an extent.

We calculated three classical measures of β-diversity (multiplicative β-diversity, proportional β-diversity and *z*-value of the species–area relationship) and three multivariate distance measures of β-diversity (mean pairwise Sørensen distance, multiple-site Sørensen distance, Hellinger's distance). We calculated:

(i) Classical multiplicative β-diversity as *γ*/*α*.
(ii) Classical proportional β-diversity as 1 − (*α*/*γ*).
(iii) *z*-value [28] of the species–area relationship as log(*γ*) − log(*α*)/log(grain number).
(iv) Mean pairwise Sørensen distance using ‘beta.pair’ function in *betapart* package in R (http://www.r-project.org/).
(v) Multiple-site Sørensen distance using ‘beta.multi’ function in *betapart* package in R.
(vi) Hellinger's distance using ‘beta.div’ function in *adespatial* package in R.

In this paper, we only present the results of classical multiplicative β-diversity because all metrics were highly correlated with each other (Pearson *r* > 0.95).

To determine if β-diversity deviated from the null expectations of random sampling (standardized β-deviation, which we refer to as β-deviation), we compared β-diversity of observed and randomized datasets [4,6]. Specifically, we generated randomized datasets by randomizing trees (greater than or equal to 1 cm DBH) across all nine grains, while retaining the relative species abundance across the extent and the total number of individuals in each grain. This accounts for variation in γ-diversity [4,6]. We generated 1000 randomized datasets for each sampling design. We calculated β-deviation = (*β*_obs_ − *β*_rand_)/s.d._rand_, where *β*_obs_ is the observed β-diversity, and *β*_rand_ and s.d._rand_ are the mean and s.d., respectively, of the expected β-diversity. Under the null hypothesis of equal values for the observed and expected β-diversity, the distribution of β-deviation is approximately standard normal [29], which we assumed when calculating *p*-values (i.e. 95% of β-deviation values are expected to fall in the range of −1.96 to 1.96) [6].

### 2.2. Latitudinal β-diversity patterns

We used tree data from 15 long-term, large-scale forest dynamics plots along a latitudinal gradient from Papua New Guinea to northern China. The Center for Tropical Forest Science/Smithsonian Institution Global Earth Observatories (CTFS/SIGEO; http://www.sigeo.si.edu/) and the Chinese Forest Biodiversity Network (CForBio; http://cfbiodiv.org/) coordinated data collections in all plots: Badagongshan, Fushan, Gutianshan, Hainan, Heishiding, Lambir, Lienhuachih, Mo Singto, Nonggang, Palanan, Pasoh, Sinharaja, Tiantongshan, Wanang, Xishuangbanna (electronic supplementary material, figure S1) [30,31]. Each of the 15 plots covers 15–52 ha of forest in which all stems with DBH of 1 cm or greater were identified and precisely mapped across the entire area.

For analyses of latitudinal β-diversity patterns, we use 20 grains of varying sizes: 10 m × 10 m (0.01 ha), 20 m × 20 m (0.04 ha), 30 m × 30 m (0.09 ha), 50 m × 50 m (0.25 ha), 70 m × 70 m (0.49 ha) and 100 m × 100 m (1 ha). We used a nested design, where we chose the first grain randomly and the remaining 19 next to each other in a 4 × 5 matrix design. We did not fit 100 m × 100 m grains into Palanan and Nonggang plots due to their small size (less than 20 ha). Extent size represents the combination of 20 grains, and therefore the extent size (γ-scale) varies with grain size (α-scale). We measured α-diversity as the mean species richness of each grain and γ-diversity as the species richness of an extent (electronic supplementary material, figure S2 and figure S3). We used the two most widely used measures of β-diversity, classical multiplicative β-diversity (*β* = *γ*/*α*) [32] and mean pairwise Sørensen dissimilarity distance, as measures of β-diversity [33]. These two metrics were highly correlated with proportional beta, *z*-value, multiple-site Sørensen and Hellinger's distance (Pearson *r* > 0.89; electronic supplementary material, figure S4).

The multiple-site Sørensen distance can be partitioned into nestedness and turnover components [34]. Nestedness represents the result of the variation in species richness, and turnover represents the result of the variation caused by species replacement [34,35]. Thus, turnover could be used as a true measure of species replacement. In this study, turnover caused *ca* 94% (range: 85–98%) of the variation and was highly correlated with multiple-site Sørensen distance at all spatial scales (Pearson *r* > 0.93). Therefore, we did not partition multiple-site Sørensen distance.

We used a randomized null-model approach to measure the deviation of observed β-diversity from the null expectations of random sampling (β-deviation; see above for details). We also calculated the rate of deviation of observed mean pairwise Sørensen from a null-model generated stochastic expectation (hereafter pairwise Sørensen deviation). We extracted mean monthly temperature and mean annual precipitation data for each plot from the WORLDCLIM database v. 1.4 [36].

## 3. Data analysis

Tree β-diversity often shows a nonlinear bi-phasic curve with spatial scale, with a faster change in β-diversity values at small spatial scales and slower change at comparatively larger spatial scales (figure 1) [19]. We therefore fitted a regression model with segmented relationships between β-diversity and spatial scale to estimate a threshold between small and large spatial scale (figure 1). Segmented regression is a method where two regression lines are fitted onto an independent variable (grain size in our analysis), which are joined together at a break point [37]. It can be used to detect changes in model fits and can be important in decision-making.

We used general linear models with normal error structure to determine the change in β-diversity and β-deviation of different grain sizes with γ-diversity and latitude. We did not include a temperature in the models as it was highly correlated with latitude (Pearson *r* = 0.9, *p* < 0.001), but precipitation was included as a covariate. We used a backward elimination technique to simplify the models. We log_e_ transformed γ-diversity prior to analysis and used absolute values of latitude. We removed the Lambir site from the models determining the change in β-deviation with varying γ-diversity and latitude because of high heteroscedasticity. Lambir had β-deviation (spatial aggregation) values up to two times higher than any other site, which may be caused by the presence of distinct soil types and strong habitat associations within this particular plot [38,39]. All analyses were conducted in the statistical program R (R Core Team, v. 3.3.1). The data are available in electronic supplementary material, appendix S1 and on request from ForestGEO (http://forestgeo.si.edu) and CForBio (http://cfbiodiv.org).

## 4. Results

### 4.1. Sensitivity of β-diversity

**Figure 2.**
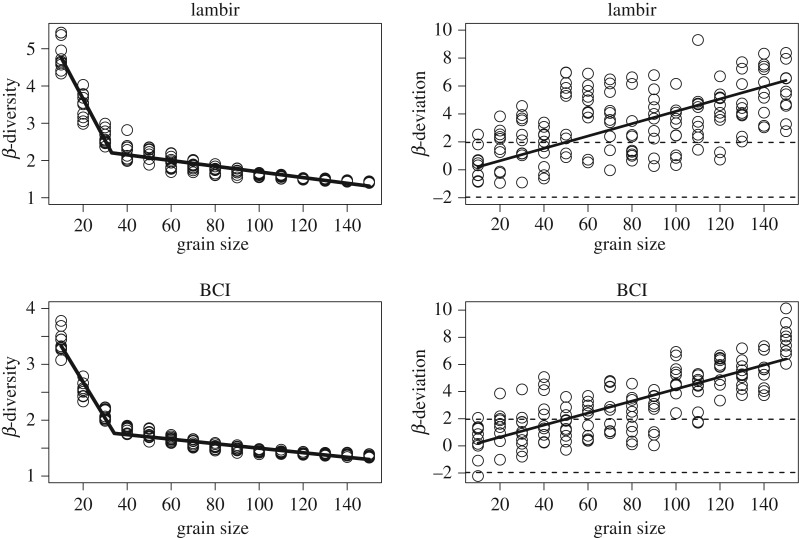
Variation in classical multiplicative β-diversity and β-deviation with increasing grain size in Lambir, Malaysia and BCI, Panama. β-deviation of zero indicates that the observed pattern does not differ from random sampling. The dashed lines in β-deviation plots represent the criterion (±1.96 s.d.) for assessing the statistical significance. The *x*-axis represents grain size at α-scale (e.g. 50 = 50 m × 50 m).

Grain size significantly influenced classical multiplicative β-diversity at both tropical forest sites with a sharp decrease in values at very small grains (Lambir: *R*^2^ = 0.94, *p* < 0.001; BCI: *R*^2^ = 0.97, *p* < 0.001; figure 2). The slope of the first segment (10 m × 10 m to 35 m × 35 m; Lambir: −0.110 ± 0.005 [s.e.]; BCI: −0.066 ± 0.003) was *ca* 16 times higher than that of the second (Lambir: −0.007 ± 0.0006; BCI: −0.004 ± 0.0003) at both sites (figure 2). Grain size had a very strong relationship with both the number of individuals sampled and γ-diversity (*R*^2^ > 0.95). For both Lambir and BCI sites, β-deviation did not differ from stochastic expectation at small grain sizes (β-deviation| < 1.96), but increased with grain size (Lambir: *R*^2^ = 0.28, *p* < 0.001; BCI: *R*^2^ = 0.63, *p* < 0.001; figure 2).

### 4.2. Latitudinal β-diversity patterns

**Figure 3.**
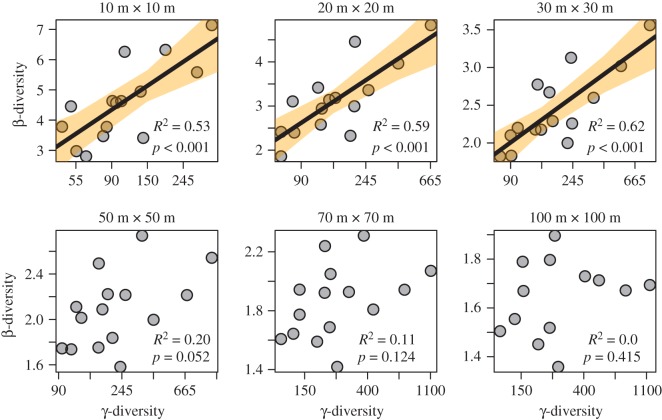
Classical multiplicative β-diversity increased with γ-diversity when sampling at small grains (10 m × 10 m to 30 m × 30 m) within each ForestGEO plot, but showed no relationship with γ-diversity at larger grains (50 m × 50 m to 100 m × 100 m).

**Figure 4.**
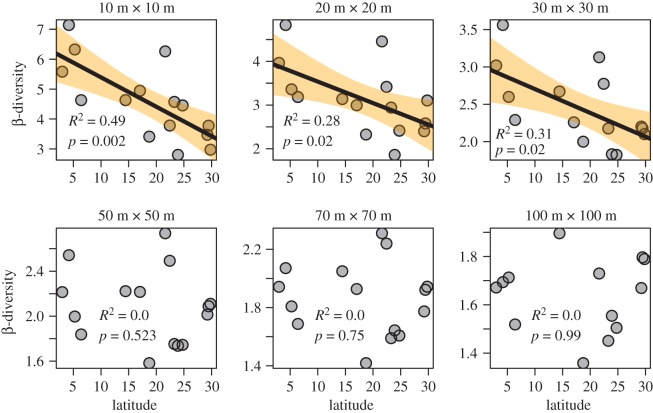
Classical multiplicative β-diversity decreased with increasing latitude when sampling at small grains (10 m × 10 m to 30 m × 30 m) within each ForestGEO plot, but showed no relationship with latitude at larger grains (50 m × 50 m to 100 m × 100 m).

Changes in precipitation did not affect either of the β-diversity metrics (classical multiplicative and mean pairwise Sørensen) at any grain size (electronic supplementary material, table S1), and precipitation was therefore eliminated from all models. Both the β-diversity metrics increased significantly with γ-diversity at small grains (10 m × 10 m to 30 m × 30 m), but showed no relationship with γ-diversity at larger grains (50 m × 50 m to 100 m × 100 m; figure 3; electronic supplementary material, figure S5). Latitudinal β-diversity patterns were similar. Both the measured β-diversity indices decreased significantly with increasing latitude while sampling at small grains (10 m × 10 m to 30 m × 30 m), but showed no relationship with latitude at relatively larger grains (50 m × 50 m to 100 m × 100 m; figure 4; electronic supplementary material, figure S5). The γ-diversity was highly correlated with α-diversity at all grain sizes (*R*^2^ > 0.84, *p* < 0.01), and the number of individuals in each grain did not change with latitude (*R*^2^ = 0.001; *p* = 0.85).

**Figure 5.**
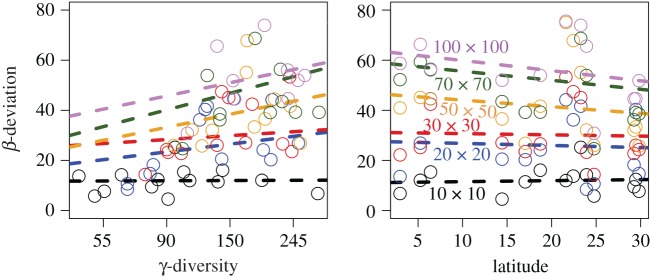
Standardized β-deviation did not vary significantly with γ-diversity and latitude at any grain size. However, β-deviation values increased significantly with grain size, indicating stronger intraspecific aggregation at larger spatial scales. Dashed lines indicate non-significant relationships.

Changes in precipitation did not affect either β-deviation or Sørensen deviation at any grain size, and so precipitation was eliminated from all models (electronic supplementary material, table S2). Standardized β-deviation did not vary with either γ-diversity or latitude at all grain sizes (figure 5; electronic supplementary material, table S3). The pairwise Sørensen deviation was similar to β-deviation. The values of pairwise Sørensen deviation also did not vary with either γ-diversity or latitude at all grain sizes (electronic supplementary material, figure S6).

## 5. Discussion

Our results demonstrate that spatial scale (grain size) changes the relationship between β-diversity and latitude. We found that β-diversity was highly dependent on γ-diversity at small grains, but not at large grains (figure 3; electronic supplementary material, figure S4). Our study therefore confirms that the use of large grains still remains to be the best-known method for measuring γ-independent β-diversity [15,40,41], unless questions specific to β-diversity at smaller spatial scales are being addressed. Their correlation is problematic because variation in γ-diversity alone can account for gradients in β-diversity [6]. At relatively large grains (greater than or equal to 0.25 ha), where β-diversity is not influenced by γ-diversity, β-diversity remained similar across the latitudinal gradient (figure 4; electronic supplementary material, figure S4).

It should be noted that the grain size is relative and will vary with sampling method and taxon. Our study sampled all trees ≥ 1 cm DBH, but when sampling trees ≥ 10 cm DBH even a grain size of 100 m × 100 m can be considered small [42]. Sampling using small grains could explain the correlation between β- and γ-diversity. For example, let us assume a homogeneous community with 100 species and a β-diversity (*β* = *γ*/*α*) of one, i.e. α-diversity is equal to γ-diversity. But, if only 40 individuals are sampled at α-scale, the probability of β-diversity being one is zero, simply caused by constraining α-diversity that makes β-diversity dependent on γ-diversity [15]. Therefore, β-diversity at small grains is higher at the equator because of sampling inadequacy, which makes it dependent on γ-diversity [6,15,20].

Methods to account for γ-dependence of β-diversity have received strong scientific attention and stirred discussions [6,7,10–12]. Previous studies used null-model generated β-deviation to account for γ-dependent effects [4,6,10]. But recent studies have challenged the use of β-deviation for comparing between habitat types [11,12], as studies that used β-deviation have resulted in contrasting conclusions within and across studies [4,6,7]. Recently, Ulrich *et al.* [12] have shown that the use of null models can result in high artificial rejection rates of focal patterns (Type II statistical errors). Our case study, along with several previous studies, suggests that the use of large grains is the best available method to avoid γ-dependence of β-diversity [15,40,41].

Our data were limited to forests in the tropics and subtropics and we did not have data from permanent plots in the temperate region (greater than 30° latitude). Recently, Castro-Insua *et al.* [43] investigated if there were any latitudinal thresholds in β-diversity, and showed that different β-diversity patterns exist on either side of a threshold at *ca* 30° latitude. Although we found no relationship between β-diversity and latitude, this relationship might change in the temperate region. Our plots also have a broader longitudinal spread that is not ideal in a study of latitudinal effects, and 7 of 15 plots are on islands. Future studies should examine latitudinal β-diversity patterns using large spatial scales in a different region that includes temperate plots. Studies using more sites, across American and African latitudinal gradients, and using multiple growth forms and larger distances between grains, will be useful to determine spatial scale effects on β-diversity patterns and differences in the mechanisms that drive community assembly.

## 6. Conclusion

Our results suggest that sampling at large sampling grains can remove the influences of γ-diversity on β-diversity. Specifically, we show that observed β-diversity does not change with increasing latitude (3–30° latitude; figure 3). Therefore, our results support the idea that β-diversity in the tropics is similar to β-diversity in the subtropics. These results have important implications for community ecology and demonstrate that the general β-diversity patterns and the processes structuring communities are still open for discussion.

## Supplementary Material

Supplementary material

## Supplementary Material

Supplementary material

## Data accessibility

Data are available from the electronic supplementary material, appendix S1, and available via CTFS-ForestGEO website at http://www.forestgeo.si.edu and CForBio website at http://cfbiodiv.org.
