# Supplementary material for: Spatial scale changes the relationship between beta diversity, species richness and latitude
Source: R Soc Open Sci. 2018 Sep 19;5(9):181168. doi: 10.1098/rsos.181168 (PMC6170539; doi:10.1098/rsos.181168)
Supplement: Supplementary material [file rsos181168supp1.docx]

**Supplementary material**

**Spatial scale changes the relationship between beta diversity, species richness and latitude**

Rachakonda Sreekar, Masatoshi Katabuchi, Akihiro Nakamura, Richard T. Corlett, J. W. Ferry Slik, Christine Fletcher, Fangliang He, George D. Weiblen, Guochun Shen^9^, Han Xu, I-Fang Sun, Ke Cao, Keping Ma, Li-Wan Chang, Min Cao, Mingxi Jiang, I. A. U. Nimal Gunatilleke, Perry Ong, Sandra Yap, C. V. Savitri Gunatilleke, Vojtech Novotny, Warren Y. Brockelman, Wusheng Xiang, Xiangcheng Mi, Xiankun Li, Xihua Wang, Xiujuan Qiao, Yide Li, Sylvester Tan, Richard Condit, Rhett D. Harrison, Lian Pin Koh

***Journal:*** *Royal Society Open Science*

Figure S1 …………………………………………………………………………………. pg. 2

Figure S2 …………………………………………………………………………………. pg. 3

Figure S3 …………………………………………………………………………………. pg. 4

Figure S4 …………………………………………………………………………………. pg. 5

Figure S5 …………………………………………………………………………………. pg. 6

Figure S6 …………………………………………………………………………………. pg. 7

Table S1 …………………………………………………………………………………... pg. 8

Table S2 …………………………………………………………………………………... pg. 9

Table S3 …………………………………………………………………………………... pg. 10


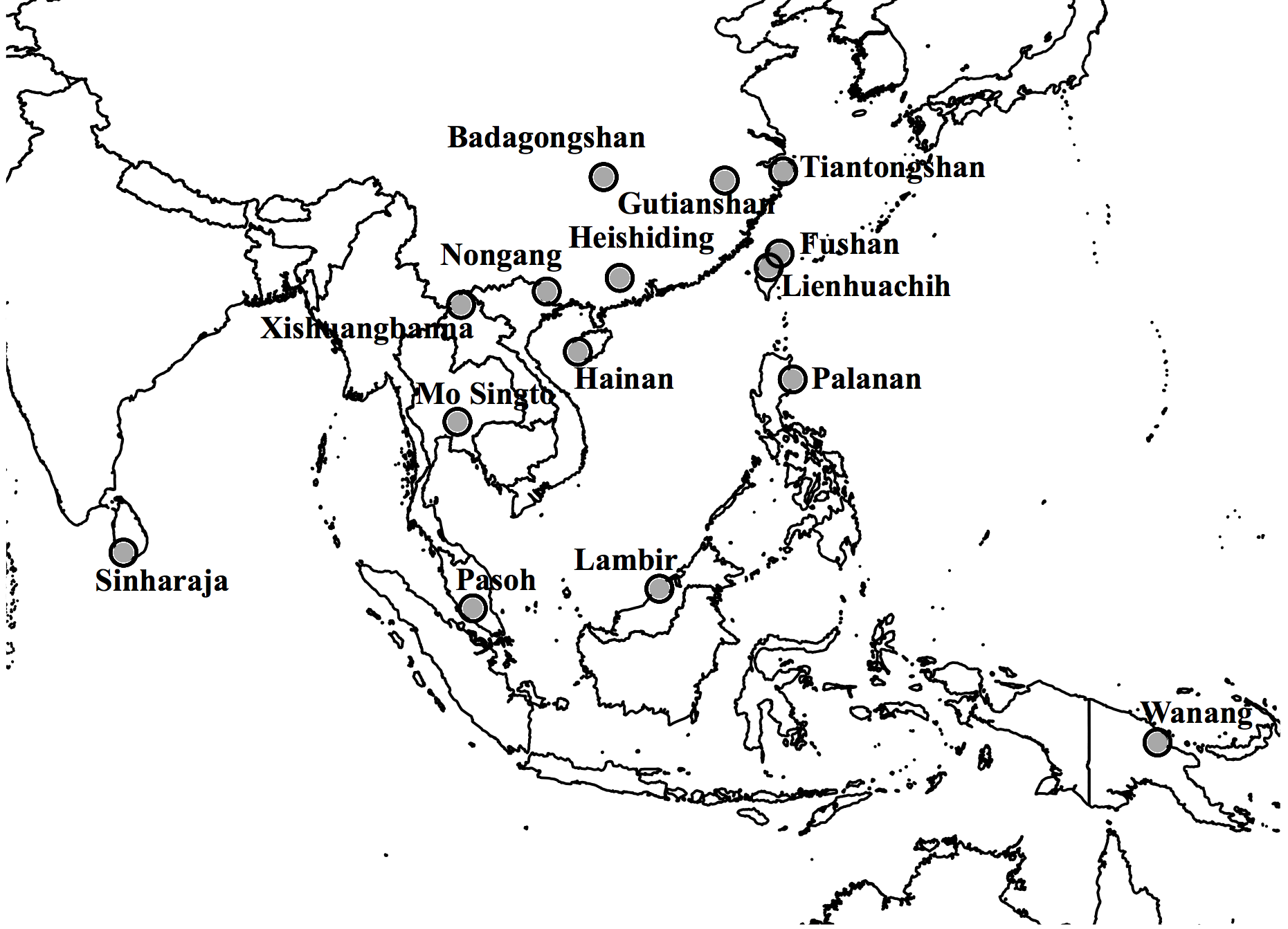


**Figure S1.** Asia-Pacific map showing the locations of the 15 large forest dynamics plots studied in this paper.

**Figure S2.** Relationship between α-diversity and grain size at all the 15 sampled sites.

**Figure S3.** Relationship between γ-diversity and grain size at all the 15 sampled sites.

**Figure S4.** Pearson’s correlation matrix of all the measured beta-diversity metrics. All measured metrics were highly correlated with each other.


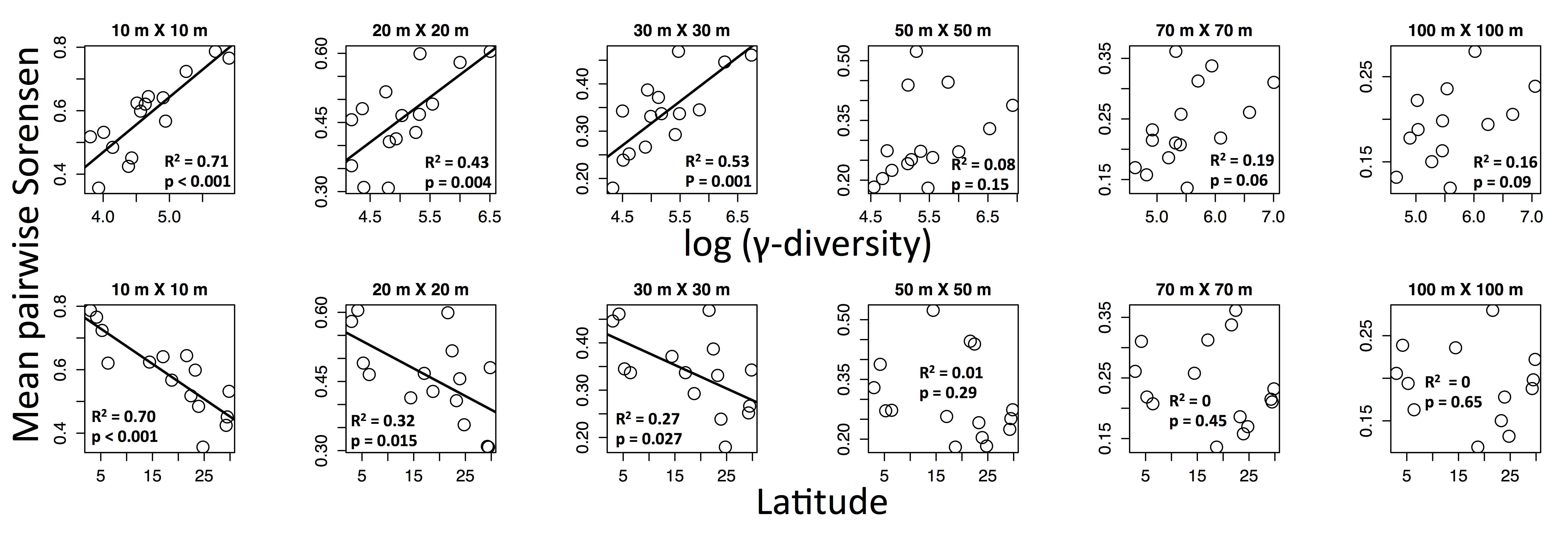


**Figure S5.** Mean pairwise Sørensen increased with γ-diversity and decreased with latitude when sampling used small grains (10 m x 10 m to 30 m x 30 m), but showed no relationship with γ-diversity and latitude at larger grains (50 m x 50 m to 100 m x 100 m).

**
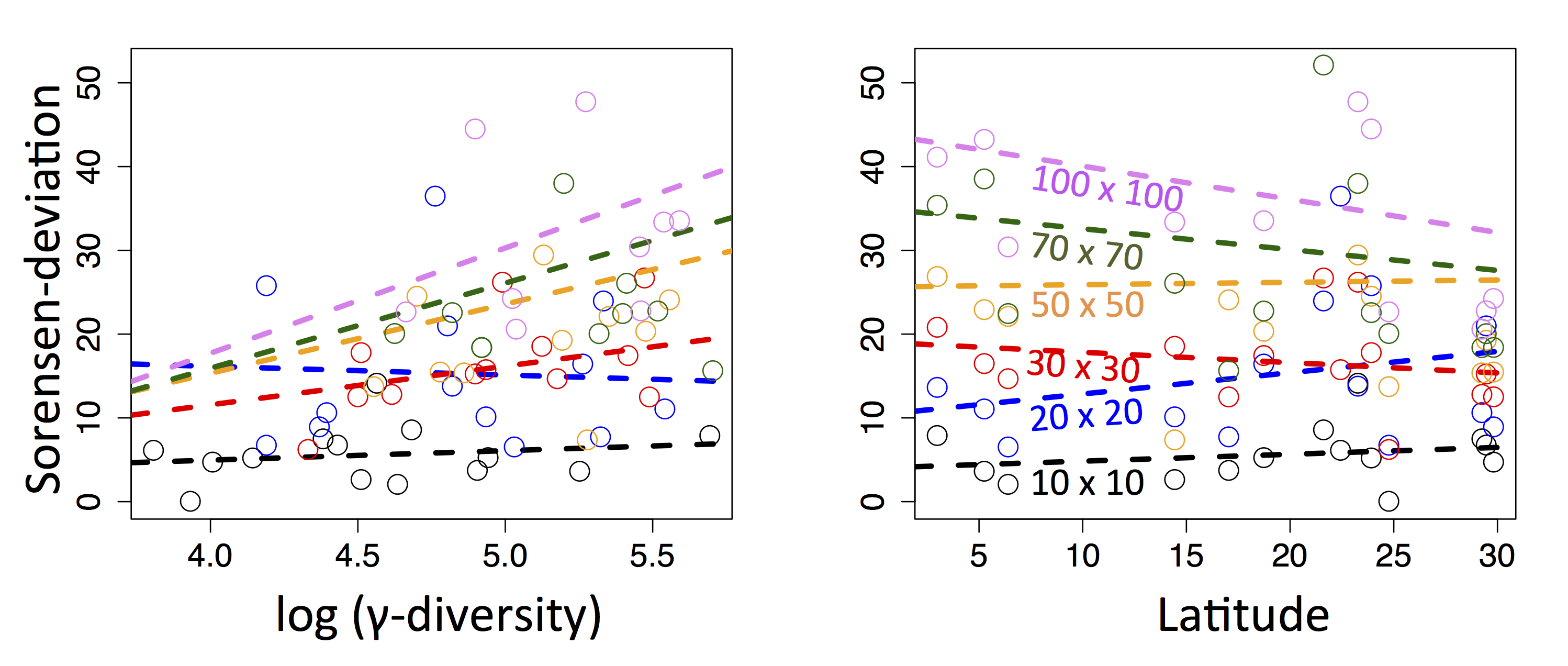
**

**Figure S6.** Standardized pairwise Sørensen-deviation did not vary significantly with γ-diversity and latitude at any grain size. However, pairwise Sørensen-deviation values increased significantly with grain size, indicating stronger intraspecific aggregation at larger spatial scales. Dashed lines indicate non-significant relationships.

**Table S1.** Relationship between precipitation and observed β-diversity metrics (classical multiplicative β-diversity and mean pairwise Sørensen)

| *Classical multiplicative β-diversity* |  |  |
| --- | --- | --- |
| **Grain size** | **Estimate ± SE** | ***P-*value** |
| 10 m x 10 m | 0.00065 ± 0.00036 | 0.096 |
| 20 m x 20 m | 0.00008 ± 0.00025 | 0.736 |
| 30 m x 30 m | 0.00000 ± 0.00016 | 0.962 |
| 50 m x 50 m | -0.00007 ± 0.0001 | 0.5 |
| 70 m x 70 m | -0.00007 ± 0.00007 | 0.352 |
| 100 m x 100 m | -0.00004 ± 0.00005 | 0.421 |
|  |  |  |
| *Mean pairwise Sørensen* |  |  |
| **Grain size** | **Estimate ± SE** | ***P-*value** |
| 10 m x 10 m | 0.00003 ± 0.00004 | 0.44 |
| 20 m x 20 m | 0.00001 ± 0.00003 | 0.594 |
| 30 m x 30 m | -0.00001 ± 0.00002 | 0.658 |
| 50 m x 50 m | -0.00004 ± 0.0003 | 0.247 |
| 70 m x 70 m | -0.00001 ± 0.00002 | 0.468 |
| 100 m x 100 m | -0.00001 ± 0.00001 | 0.46 |

**Table S2.** Relationship between precipitation and null-model generated β-deviation and Sørensen-deviation

| *β-deviation* |  |  |
| --- | --- | --- |
| **Grain size** | **Estimate ± SE** | ***P-*value** |
| 10 m x 10 m | -0.00021 ± 0.00172 | 0.901 |
| 20 m x 20 m | -0.00125 ± 0.00423 | 0.772 |
| 30 m x 30 m | -0.00306 ± 0.00372 | 0.425 |
| 50 m x 50 m | -0.00366 ± 0.00468 | 0.467 |
| 70 m x 70 m | -0.00362 ± 0.00638 | 0.581 |
| 100 m x 100 m | -0.00106 ± 0.00446 | 0.817 |
|  |  |  |
| *Sørensen-deviation* |  |  |
| **Grain size** | **Estimate ± SE** | ***P-*value** |
| 10 m x 10 m | -0.00206 ± 0.00095 | 0.052 |
| 20 m x 20 m | -0.00434 ± 0.00256 | 0.116 |
| 30 m x 30 m | -0.00246 ± 0.00162 | 0.156 |
| 50 m x 50 m | -0.00349 ± 0.00528 | 0.52 |
| 70 m x 70 m | -0.00395 ± 0.00518 | 0.46 |
| 100 m x 100 m | -0.00098 ± 0.00512 | 0.851 |

**Table S3.** Latitude and γ-diversity did not affect standardized β-deviation across all sampling scales (garin sizes).

|  | γ-diversity | | Latitude | |
| --- | --- | --- | --- | --- |
| **Quadrat size** | **R^2^** | ***P-value*** | **R^2^** | ***P-value*** |
| 10 m x 10 m | 0.001 | 0.95 | 0.004 | 0.81 |
| 20 m x 20 m | 0.03 | 0.53 | 0.009 | 0.75 |
| 30 m x 30 m | 0.002 | 0.87 | 0.01 | 0.74 |
| 50 m x 50 m | 0.07 | 0.37 | 0.07 | 0.38 |
| 70 m x 70 m | 0.06 | 0.40 | 0.06 | 0.41 |
| 100 m x 100 m | 0.16 | 0.21 | 0.15 | 0.23 |
